# Temporal Distribution of Imidacloprid and Its Metabolites in Laying Hens: An Evaluation of the Potential Impact on Food Safety

**DOI:** 10.1021/acs.jafc.4c08378

**Published:** 2025-05-02

**Authors:** Mayra F. Tsoi, Justin Zyskowski, Cara Robison, Andreas F. Lehner, Levent Dirikolu, John P. Buchweitz

**Affiliations:** † Department of Pathobiology and Diagnostic Investigation, College of Veterinary Medicine, Michigan State University, 784 Wilson Rd, East Lansing, Michigan 48824, United States; ‡ Veterinary Diagnostic Laboratory, Michigan State University, 4125 Beaumont Rd, Lansing, Michigan 48910, United States; § Department of Animal Sciences, Michigan State University, 474 Shaw Lane, East Lansing, Michigan 48824, United States; ∥ Department of Comparative Biomedical Sciences, School of Veterinary Medicine, Louisiana State University, Skip Bertman Dr, Baton Rouge, Louisiana 70803, United States

**Keywords:** neonicotinoid, pesticide, poultry, LC-MS/MS

## Abstract

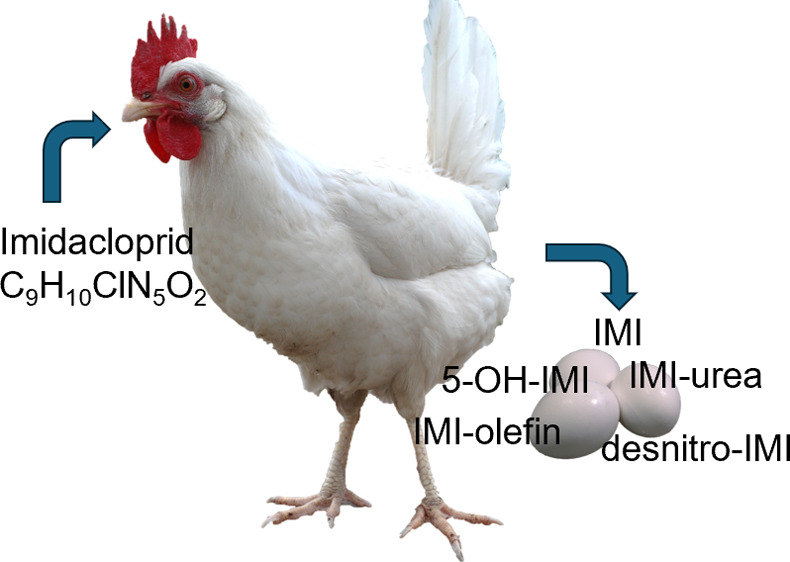

Imidacloprid (IMI) is widely used in poultry houses in the United States to control darkling beetles. However, there is limited knowledge regarding the persistence and distribution of IMI and its metabolites in poultry products following acute exposure to subclinical concentrations. In this study, mature hens received a dose of imidacloprid (placebo, 1 mg/kg, or 10 mg/kg), and liquid chromatography tandem mass spectrometry was used to quantify IMI and its metabolites in tissues. IMI was below the limit of quantitation in plasma within 24 (1 mg/kg) and 48 h (10 mg/kg). At 10 mg/kg, IMI equivalents (sum of IMI and its metabolites) exceeded the regulatory threshold as established by the United States Code of Federal Regulations for pectoral and thigh muscle, brain, liver, spleen, kidney, fat, and eggs after 24 h. At 1 mg/kg, IMI equivalents surpassed the threshold for liver, spleen, and eggs after 24 h. These results suggest that low dose exposure to IMI may cause poultry products destined for human consumption to violate federal regulations.

## Introduction

Darkling beetles (*Alphitobius diaperinus*) are endemic to poultry houses, spending their life cycle within wet litter. They cause structural damage to the houses (including insulation), damage the quality of carcasses (e.g., by biting birds), decrease food consumption, and most importantly, serve as vectors for avian pathogens. These pathogens include avian influenza virus, Marek’s disease virus, infectious bursal disease virus, and foodborne pathogens such as *Salmonella* sp. and *Escherichia coli*.^1−3^ In one recent study, 69 days after SPF leghorn chickens that were orally inoculated with 10^8^*Salmonella enteritidis* were removed from containers, Salmonella was still cultured from beetles, demonstrating its ability to serve as a vector for infecting the next flock of birds.^4^ Given its wide-ranging effects on buildings and birds, the economic impact of darkling beetle infestation alone is nearly impossible to assess. Imidacloprid N-(1-[6-chloro-3-pyridinyl)methyl]-4,5-dihydroimidazol-2-yl]nitramide; IMI) is a neonicotinoid pesticide widely used in U.S. poultry houses, primarily for broilers, to control darkling beetle larvae and adults. Its recommended use is as a diluted spray for treating surfaces (walls, beams, and floors) and litter (if infestation is severe) in poultry houses in between flocks.^5^ It is important to note that, unlike the United States and with few emergency exceptions, imidacloprid is banned from many agricultural practices in the European Union.^6^

IMI’s method of action is to selectively bind and overactivate nicotinic acetylcholine receptors in the central nervous system of insects, leading to seizures, paralysis, and death.^7^ Due to its lower binding affinity for vertebrate nicotinic receptors, its use in vertebrates has historically been considered of lower risk, if used according to label instructions.^7,8^ However, in cases of suspected IMI toxicosis in birds, the birds have often consumed dead beetles, large amounts of treated litter, or litter contaminated by an IMI spill. For example, a mixing error resulted in high mortality and neurologic signs in a flock of 27,000 four-day-old broilers within 48 h of placement.^9^ It was surmised that these birds likely ingested concentrated product from the walls.^9^ Its mechanisms of toxicity are numerous and involve production of reactive oxygen species, DNA damage, apoptosis, and lipid peroxidation.^10^

In Japanese quail, IMI has been shown to be rapidly absorbed into the bloodstream within 1 h postingestion, distributed to the brain, muscle, kidney, and liver, metabolized primarily to 5-hydroxyimidacloprid (5-OH-IMI) in the liver, and excreted rapidly in feces within 24 h.^7^ In rock pigeons, IMI residues were detected in the liver, pectoral muscle, brain, and kidney 24 h after repeated oral dosing of IMI for 4 weeks.^11^ Similarly, IMI and its metabolites, 5-OH-IMI and imidacloprid-olefin (IMI-olefin), have also been detected in feces, liver, muscle, spleen, kidney, and brain of domestic chickens 15 days after the last exposure to IMI following daily exposure for 7 days.^8^

The specific absorption, distribution, metabolism, and excretion profile of IMI in laying hens and the presence of IMI and its metabolites in eggs are unknown. Therefore, the primary goal of this study was to evaluate food safety in the context of IMI’s temporal distribution and metabolite profile. To achieve this, IMI and its 6-chloropyridinyl moiety-containing metabolites (IMI-urea, IMI-olefin, desnitro-IMI, 5-OH-IMI, and 6-chloronicotinic acid) were quantitatively measured in blood, tissue, feces, and eggs following a single subclinical dose over time. These concentrations were measured using a liquid chromatography tandem mass spectrometry (LC-MS/MS) method and compared with tolerances established in the Code of Federal Regulations (CFR) and Article 12 of the European Food Safety Authority (EFSA) Regulation No 396/2005.^12,13^

## Materials and Methods

### Animals and Housing

Seventy-five, 61-week-old, Hy-line brown hens weighing 1.6–2.4 kg were housed in individual, 22” × 24” × 16” cages with nest box curtains and scratch pads, on two levels. Hens were acclimatized for 1 week on a 16-h light:8-h dark cycle. A wall exhaust fan maintained the temperature between 17–19 °C. A standard layer diet containing corn and soybean meal, 4.9% calcium and 16.5% protein was available *ad libitum* (Webberville Feed & Grain Co., Webberville, MI, USA). Water was available *ad libitum*. Hens were weighed and randomly assigned to one of three treatment groups (*n* = 5 hens/group) that received imidacloprid at 1 and 10% of a reported LD50 for chickens corresponding to 1 and 10 mg/kg of IMI or a placebo treatment (Lafeber Nutristart 1:3 in water; Lafeber Co., Cornell, Il, USA).^14^ Birds were fasted overnight. Prior to gavage, crops were palpated to ensure that they were empty.

### Treatment and Sampling

Analytical grade (active) imidacloprid powder (ChemService, West Chester, PA, USA) was dissolved in ethanol (10% v/v), mixed with corn oil, and then further dissolved in Lafeber Nutristart (1:3 in water; Lafeber Co., Cornell, Il, USA) to obtain final concentrations of 0.8 or 0.08 mg/mL.^8,14^ Treatments were prepared on the morning of administration, protected from light, kept on ice, and mixed thoroughly prior to administration. The concentrations and purity of IMI were verified before the start of the trial. IMI was administered by oral gavage using a 60-cc syringe and a 10 Fr × 16 in. red rubber urethral feeding catheter. After each administration, the catheter was flushed with 5 mL of water. Birds received a similar volume of treatment or placebo (20–30 mL).

Approximately 2–3 mL of blood was sampled from the brachial vein at 1, 2, 4, 24, 48, and 168 h postexposure, transferred into 2 mL EDTA tubes, sat at room temperature for 1–2 h, centrifuged at 1200*g* for 10 min, plasma was transferred into microcentrifuge tubes, and samples were kept at 4 °C until analysis within 1 week of study completion. Birds were humanely euthanized by cervical dislocation (*n* = 5 hens/treatment/time point). At 1, 4, 24, 48, and 168 h postexposure, approximately 1.0 g from the left pectoralis major, left quadriceps, left liver lobe, coelomic fat pad, spleen, right cranial and middle kidney lobes, and brain (cerebrum, optic lobes, cerebellum, brainstem) were collected, placed in Whirl-Pak sample bags (Nasco Sampling LLC, Madison, WI, USA), and kept at −20 °C until analysis. Whole eggs that were spontaneously laid or near completion in the shell gland were collected at 24 (*n* = 27), 48 (*n* = 24), 72 (*n* = 13), and 168 h (*n* = 12) after treatment and cracked in half, and yolk and albumen were kept in screw top specimen containers at −20 °C until analysis. Feces (cloacal droppings and urates) were collected 24, 48, and 168 h postexposure in Whirl-Pak sample bags and kept at −20 °C until analysis. Doses and time points were based on similar studies done in chickens^8^ and Japanese Quail.^7^ All animal procedures were approved by the Institutional Animal Care and Use Committee (IACUC) at MSU no. PROTO202300371.

### Liquid Chromatography Tandem Mass Spectrometry

Chemical standards of imidacloprid, *d*4-imidacloprid, 5-hydroxyimidacloprid, 2-imidazolidone, 6-chloronicotinic acid, 6-hydroxynicotinic acid, desnitro imidacloprid, imidacloprid urea, and imidacloprid olefin were obtained from Sigma-Aldrich (Sigma-Aldrich, St. Louis, MO, USA). Acetonitrile and formic acid were purchased from Sigma-Aldrich. Ultrapure water used for the mobile phases was prepared by a Milli-Q Millipore Water System.

A stock solution containing all 8 target analytes was prepared at 100 ng/mL in acetonitrile. Working standards were prepared via serial dilutions to concentrations 0.05, 0.1, 2.5, 5, 7.5, 10, 25, and 50 ng/mL with internal standard (ISTD) imidacloprid-*d*4 at a concentration of 0.5 ng/mL.

Tissues were prepared for analysis by sectioning 0.5 g of homogeneous tissue (free of connective and vascular tissue) and placing it in a 7 mL Precellys bead tissue homogenization tube. Egg yolk and albumin from individual eggs were homogenized with an Omni International (Kennesaw, GA, USA) 125/TM125-11 hand-held homogenizer with a stainless steel 7 mm generator probe. Similar to tissues, 0.5 g of egg homogenate was placed in a 7 mL Precellys bead tissue homogenization tube. Two milliliters of acetonitrile containing 1 ng/mL of ISTD was added. Tissues were homogenized at room temperature with a Precellys Evolution Homogenizer (Bertin Technologies, Montigny-Le-Bretonneux, France): Speed–4500*g*, Cycle 2 × 20 s with a 20 s pause between cycles. The homogenized tissues and egg were centrifuged at 5400*g* for 10 min to pellet solid material at the bottom of the tube. 250 μL of the clear top layer from each tube was transferred to an autosampler vial for LC-MS/MS analysis. If there was visible material in the top layer, then the solution was filtered through a 0.22 μm PTFE membrane before transferring to the analytical vial.

Neonicotinoids were extracted from 200 μL of plasma, which was added to a test tube with 400 mL of 0.5 ng/mL ISTD in acetonitrile. The mixture was vortexed for 40 s until well-mixed and then centrifuged for 5 min at 3000*g*. The supernatant was removed and filtered through a 0.2 μm PTFE membrane filter into an autosampler vial for LC-MS/MS analysis.

Analysis was performed with a liquid chromatographic-tandem mass spectrometer, an AB Sciex 6500+ ESI-MS/MS (SCIEX, Framingham, MA, USA) interfaced to a Shimadzu LC (Shimadzu Corp, Kyoto, Japan). Chromatography was achieved using the Kinetex F5:2.6 μm (100 mm × 3 mm) column (Phenomenex, Torrance, CA, USA). The column temperature was maintained at 40 °C with a column heater. Injection volume was 10 μL. The mobile phase consisted of 0.1% formic acid in Milli-Q water (mobile phase A) and 0.1% formic acid in acetonitrile (mobile phase B) at a flow rate of 0.5 mL/min. The mobile phase gradient is provided in Table 1. The electrospray source optimization is given in Table 2. Quantifying multiple reaction monitoring (MRM) transitions included: imidacloprid 256.1 → 209.3; 5-hydroxyimidacloprid 273.0 → 226.0; 2-imidazolidone 87.1 → 44.1; 6-chloronicotinic acid 158.1 → 122.1; 6-hydroxynicotinic acid 140.2 → 122.0; desnitro imidacloprid 212.0 → 99.0, imidacloprid urea 212.1 → 128.2; and imidacloprid olefin 254.0 → 206.0. The test was linear between 0.05 and 50 ng/mL. The limit of quantitation for all compounds was set to 0.5 ng/mL, as this is 1% of the threshold of concern.

**Table 1:** Liquid Chromatography Gradient Program

**time (min)**	**%A**	**%B**
0.1	85	15
0.5	85	15
5.5	65	35
6	5	95
6.5	5	95
7	90	10

**Table 2:** Electrospray Ionization Source Optimization

curtain gas	45
collision gas	9
ion spray voltage	5500
temperature	550
ion source gas 1	45
ion source gas 2	40

Data analysis was performed by confirming the identity of each compound analyzed by the retention time of the peak and corresponding MRM ion transitions specific to each compound. The ratio of the peak area of the largest MRM transition of each compound to that of the ISTD was calculated and fit to a standard curve to calculate the final concentration of each component in the original tissue.

Graphs were created using GraphPad Prism (GraphPad Software, Version 10.2.3, Boston, MA, USA).

## Results

### Validation of Quantitative Procedures for Imidacloprid and Its Metabolites

Limits of detection (LOD) and quantitation (LOQ) were calculated by the method of evaluation of calculated responses for peaks found in blank runs.^15^ LOD/LOQ values in ng/g were calculated as follows: imidacloprid: 0.023/0.069; imidacloprid urea: 0.017/0.050; imidacloprid olefin: 0.035/0.105; 6-chloronicotinic acid: 0.013/0.039; 6-hydroxynicotinic acid: 0.053/0.160; desnitro-imidacloprid: 0.744/2.254; 5-OH imidacloprid: 0.341/1.032. Linearity was assessed on the basis of the coefficient of correlation (*R*^2^) for least-squares-derived standard curve fits generated by the Sciex software, and average values (*n* = 3) all exceeded 0.9950 and were specifically as follows: imidacloprid: 0.99681 ± 0.00124; imidacloprid urea: 0.99851 ± 0.00058; imidacloprid olefin: 0.99798 ± 0.00070; 6-chloronicotinic acid: 0.99950 ± 0.00041; 6-hydroxynicotinic acid: 0.99608 ± 0.00202; desnitro-imidacloprid: 0.99863 ± 0.00007; 5-OH imidacloprid: 0.99837 ± 0.00044. Analyte carryover on the instrument was determined by measuring the concentration corresponding to any identified peak in a blank run following the highest calibrator (50 ng/g), and carryover was very minimal, ranging from 0.0 to 0.09%. Specific carryover values were: imidacloprid: 0.029% ± 0.025%; imidacloprid urea: 0.026% ± 0.030%; imidacloprid olefin: 0.058% ± 0.080%; 6-chloronicotinic acid: 0.090% ± 0.100%; 6-hydroxynicotinic acid: 0.017% ± 0.020%; desnitro-imidacloprid: none detected; 5-OH imidacloprid: none detected.

Precision of the analysis was determined by constructing polynomial standard curves in MS Excel (Microsoft, Redmond, WA, USA) from individual standard curve data and calculating the average of backfit values on each compound’s standard curve, the corresponding standard deviation (*n* = 3), and %RSD as percent standard deviation/average. This provided intraday precision and was determined for calibrators at 0.05, 0.10, 0.25, 0.50, 1.0, 2.5, 5.0, 7.5, 10.0, 25.0, and 50.0 ng/g. Interday precision was simply the average results across precision calculations done on three nonconsecutive days. The calculated %RSDs remained below an acceptable 5% until the lowest calibrators of 0.05, 0.1, 0.25 ppb where values became less predictable; this is a result of the Horwitz Horn effect,^16^ where lower concentrations result in increased uncertainty. By this determination, standard curves were deemed acceptable with the following lower limits of quantitation: (1) imidacloprid, 0.1 ppb; (2) 6-hydroxynicotinic acid, 0.25 ppb; (3) imidacloprid urea, 0.25 ppb; (4) 6-chloronicotinic acid, 0.05 ppb; (5) imidacloprid olefin, 0.25 ppb; (6) desnitro-imidacloprid, 1 ppb; and (7) 5-OH imidacloprid, 1 ppb. Day-to-day standard curve reliability or stability was also assessed by applying raw area counts to the first standard curve in a series of 9 totals for each compound. Precision measured in this manner showed: (1) imidacloprid, 6-hydroxynicotinic acid, and imidacloprid urea were reliable to 0.1 ppb up to 3 days; (2) 6-chloronicotinic acid was reliable to 0.05 ppb up to 3 days; (3) desnitro-imidacloprid was reliable to 0.4 ppb up to 3 days; and (4) imidacloprid olefin and 5-OH imidacloprid were reliable to 1 ppb up to 3 days.

Accuracy was assessed as refit values of standards on individual standard curves as calculated by the Sciex software, by MS Excel on each standard’s manually constructed individual curve, and by MS Excel using the first Sciex-created curve across 9 standard curve values. Five of the six compounds (imidacloprid, 6-hydroxy-nicotinic acid, imidacloprid urea, 6-chloronicotinic acid, and imidacloprid olefin) showed accuracies exceeding 100% for the 25 and 50 ppb standards when calculated by the Sciex software, in contrast to Excel using either individual curves or the first standard curve, which gave reasonable refit values for those high standards. In contrast, the Sciex software performed better at refit of lower values (0.5 ppb and below), where Excel gave recovery values too high or too low. These patterns are a consequence of two factors: (1) constraining the standard curves to linear relationships in the Sciex software, which skews values at the high end and (2) the inability of Excel software to provide 1/*x*-weighted standard curves at the low end, which skews low values despite applying polynomial equations. Although linear standard curves gave *R*^2^ values >0.995, polynomial curve fitting of the data exceeded 0.998–0.999 for all standard curves.

Specificity was investigated in detail to ensure that the target compounds could be well distinguished from one another as well as from background components. Retention time comparison for the compounds shows possible overlap for 6-chloronicotinic acid (2.426 min) and imidacloprid urea (2.384 min). Concerns in this area were resolved by examining the distinguishing effects of precursor ion choices for each compound. Qualifier and quantifier MRM precursor → product ion relationships for each compound were all highly distinguishable from one another and therefore highly specific. The qualifier/quantifier area ratios gave specific average values for each compound, with low standard deviations averaging ∼0.3. Area ratio stability was reasonably stable over nine readings over 3 days for each compound. Area ratios were also reasonably stable across the concentration range of 0.05–50 ppb for all the compounds. For transparency, the stability skews at the following concentrations for each compound: (1) imidacloprid, < 0.05 ppb; (2) 6-hydroxynicotinic acid, 0.1 ppb; (3) imidacloprid urea, 0.05 ppb; (4) 6-chloronicotinic acid, 0.05 ppb; (5) imidacloprid olefin, 0.05 ppb; (6) desnitro-imidacloprid, < 0.05 ppb; and (7) 5-OH imidacloprid, < 0.05 ppb. Note that it is acceptable for relative abundances of diagnostic ions to vary within a 40% relative abundance window (±20%) according to the Association of Official Racing Chemists.^17^ Potential interferences were assessed by screening of principal mass spectral *m*/*z* values in the Wiley Mass Spectral Browser (John Wiley & Sons, Inc., Hoboken, NJ, USA) and from a list of 19 candidate compounds; only 2-chloroniconicotinamide may require consideration as a possible interferent. The parameters for these imidacloprid compounds’ data acquisition are considered validated. Sample extraction approaches to maintain low quenching of the signal by matrix background components are considered validated by reference to the work of Selahle et al.^18^ This group reviewed extraction procedures, including liquid–liquid, solid-phase, dispersive liquid–liquid microextraction, dispersive solid phase extraction (DSPE), magnetic SPE, and cloud point extraction as examples of unique extraction methods.

Percent recoveries for each analyte in the varied tissue matrices tested (Supp. Table 1) were calculated across the range of matrix matched standards. Recoveries were between 80 and 120% for most metabolites across all tissue types. Several instances of excess recoveries for 5-OH-IMI and 6-OH-IMI were observed; however, these excesses primarily occurred at the 1 to 2 ng/g concentration where higher measurement error is more likely.

### Temporal Distribution

**Figure 1:**
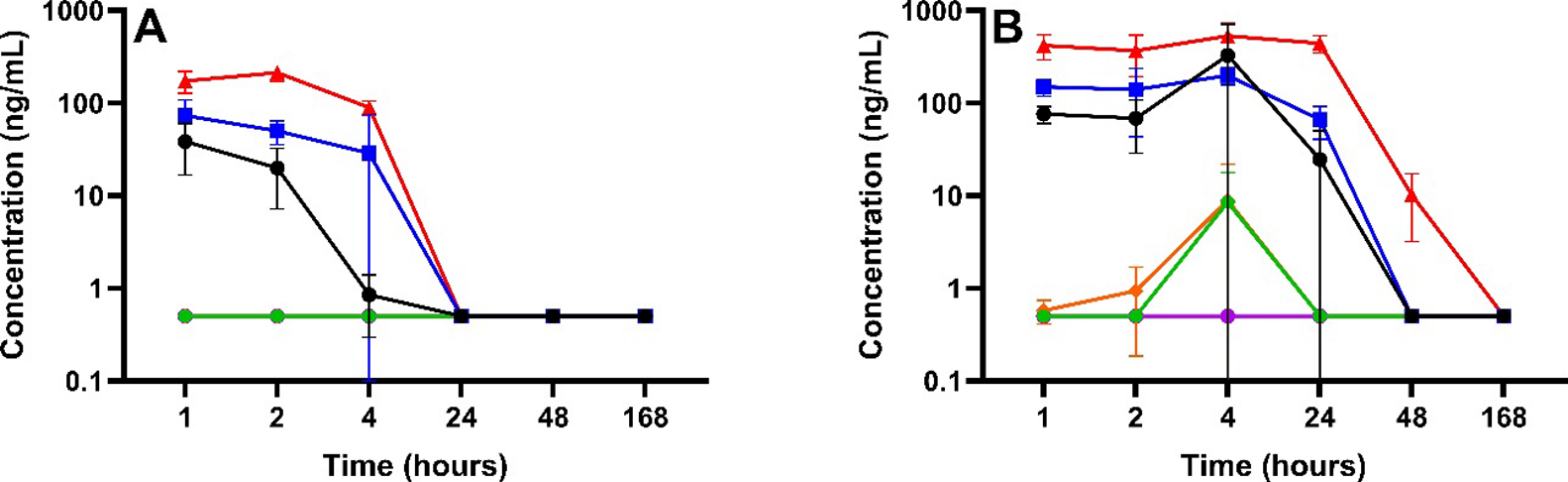
Concentration versus time profile of IMI and its 6-chloro-pyridinyl moiety metabolites in plasma for (A) 1 mg/kg treatment group and (B) 10 mg/kg treatment group. IMI (black), 5-OH-IMI, (blue), IMI-olefin (red), IMI-urea (orange), desnitro-IMI (green), and 6-CNA (purple) were measured in plasma by LC-MS/MS to an LOQ of 0.5 ng/mL (ppb). Results are presented as the mean ± standard deviation.

In plasma, IMI and its metabolites had mostly reached peak concentrations by 1 h for the 1 mg/kg treatment group, with concentrations of IMI and metabolites (5-OH-IMI and IMI-olefin) dropping below the LOQ by 24 h (Figure 1A). Peak concentrations of IMI and its metabolites (5-OH-IMI, IMI-olefin, desnitro-IMI, and IMI-urea) were observed at 4 h postdosing for the 10 mg/kg treatment group (Figure 1B). Neither IMI nor its metabolites were detected in the placebo group for any sample type (data not shown).

IMI distributed to all tissues tested including muscle (pectoral and thigh) (Supp. Figure 1), adipose tissue (Supp. Figure 2), brain, liver, spleen, kidney (Supp. Figure 3), and egg (Supp. Figure 4). The IMI distribution for the 10 mg/kg treatment group 1 h postexposure presented with the following pattern: Brain (217.2 ± 168.0 ng/g) > Muscle [Pectoral (118.8 ± 139.6 ng/g) > Thigh (49.6 ± 38.5 ng/g)] > Egg (39.5 ± 30.4 ng/mL) > Fat (35.7 ± 19.8 ng/g) > Spleen (21.7 ± 7.4 ng/g) > Liver (19.9 ± 8.0 ng/g) > Kidney (2.5 ± 1.5 ng/g). The pattern of distribution for the 1 mg/kg treatment group at the same time point was: Brain (7.2 ± 3.4 ng/g) > Spleen (6.3 ± 3.5 ng/g) > Fat (5.0 ± 5.9 ng/g) > Liver (2.2 ± 1.2 ng/g) > Muscle [Thigh (1.8 ± 0.9 ng/g) > Pectoral (<0.5 ± 0.0 ng/g)] = Egg (<0.5 ± 0.0 ng/g) = Kidney (<0.5 ± 0.0 ng/g).

In this study, IMI was metabolized predominantly to 5-OH-IMI and IMI-olefin, with IMI-olefin representing the more abundant metabolite observed in most tissues except in the kidney where it was notably absent (Supp. Figures 1–4). IMI-urea was present in the 10 mg/kg treatment group for all sample types except in the liver. IMI-urea was equally abundant in kidney with 5-OH-IMI (Supp. Figure 3). Additionally, there was evidence in fecal matter for the formation of 6-hydroxy nicotinic acid (6-OH-NA), a minor photolysis product of IMI found in water and major degradation product found in soil (Supp. Figure 5).^19^ There was no evidence of this degradation product in any of the other analyzed tissues.

### Response to Treatment and Egg Production

**Figure 2:**
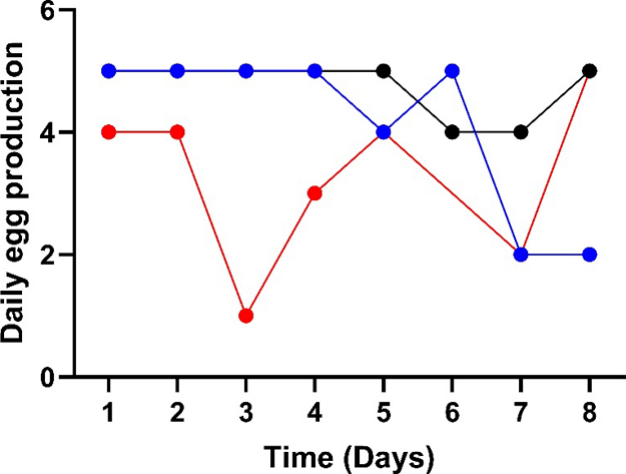
Daily egg production. Egg counts are presented for the dosing of poultry at 0 mg/kg IMI control (blue), 1 mg/kg IMI (black), and 10 mg/kg IMI (red); *n* = 5 hens per group.

Birds were closely monitored postgavage for any adverse reactions. No birds showed any neurologic signs. Daily egg production (Figure 2) was initially marginally reduced in the 10 mg/kg treatment group with a marked reduction (80%) evident by 3d post-treatment and returning to baseline by day 5. There were some late study fluctuations in egg production for the control and both treatment groups.

### Food Safety

#### Meat

**Figure 3:**
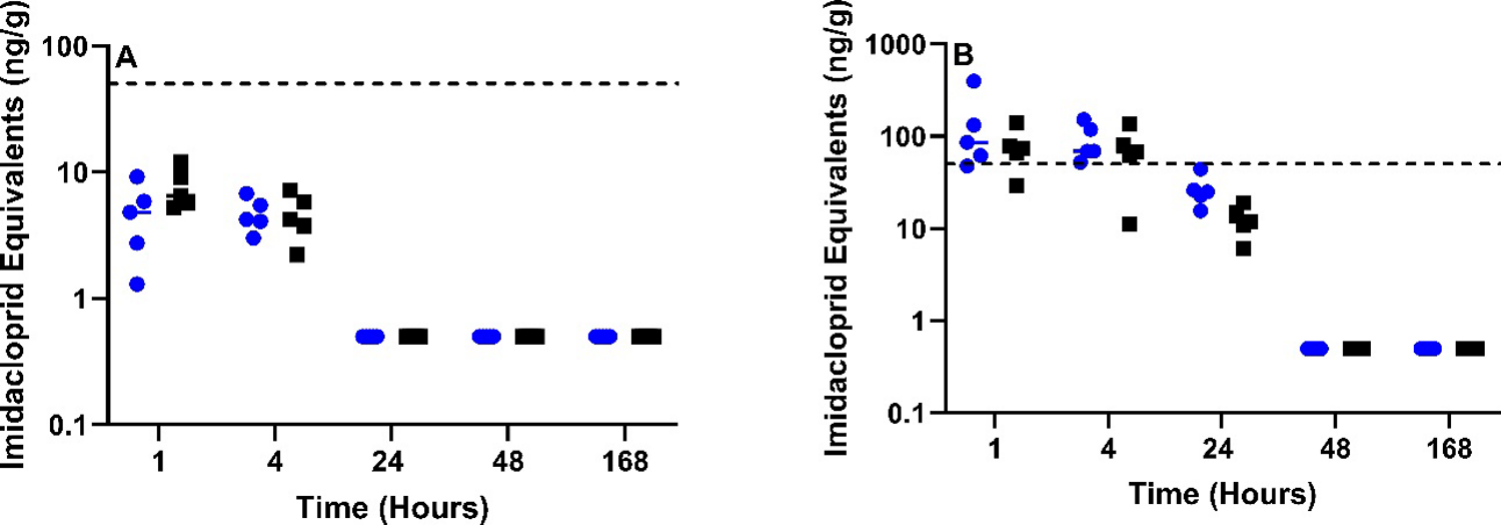
Sum of IMI and molar equivalent concentrations of 6-chloropyridinyl moiety metabolites for poultry meat. IMI dosing of poultry at (A) 1 mg/kg and (B) 10 mg/kg. IMI equivalent concentrations were calculated for pectoral (blue) and thigh (black) muscle. The dashed line represents the EFSA 396/2005 and 40 CFR § 180.472 tolerance thresholds for poultry meat (50 ng/g).

Pectoral and thigh muscle were evaluated for IMI equivalents based on definitions established in the CFR^12^ and EFSA.^13^ Although IMI equivalents were present in the 1 mg/kg treatment group, they did not exceed the diagnostic threshold of concern (50 ppb) (Figure 3A). Both muscle types, however, were in violation of the CFR at both 1- and 4 h post-treatment in the 10 mg/kg group (Figure 3B).

#### Poultry Byproducts

**Figure 4:**
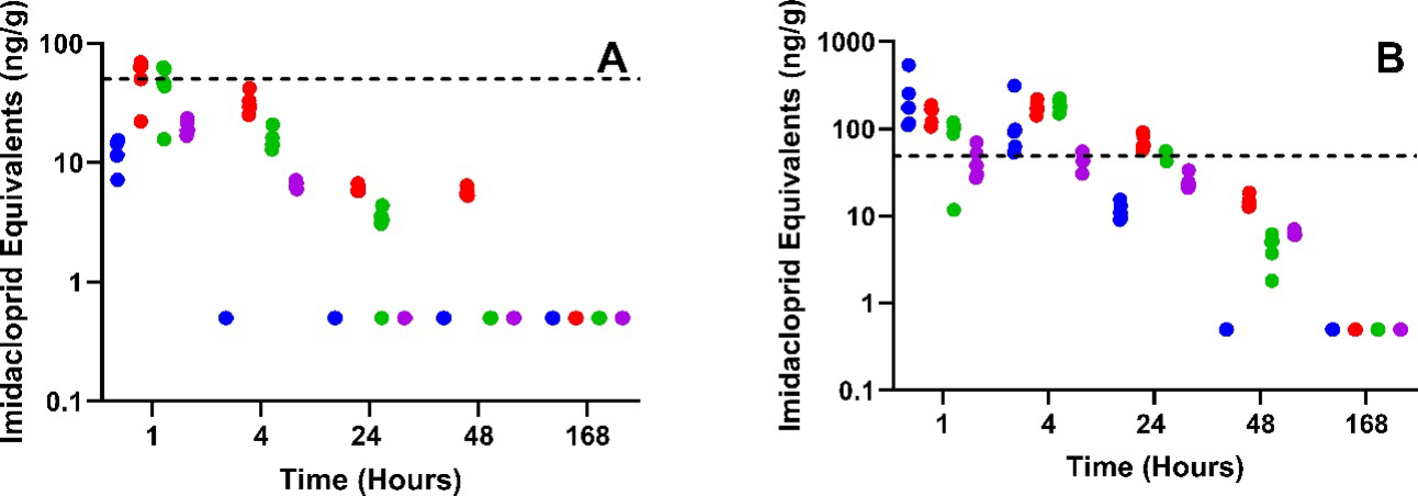
Sum of IMI and molar equivalent concentrations of 6-chloropyridinyl moiety metabolites for poultry meat byproducts. IMI dosing of poultry at (A) 1 mg/kg and (B) 10 mg/kg. IMI equivalent concentrations were calculated for brain (blue), liver (red), spleen (green), and kidney (purple) tissue. The dashed line represents the EFSA 396/2005 and 40 CFR § 180.472 tolerance thresholds for poultry meat byproducts (50 ng/g).

Brain, liver, spleen, and kidneys were evaluated individually for IMI equivalents. Liver and spleen both exceeded the 50 ppb (50 ng/g) threshold at 1-h post exposure in the 1 mg/kg treatment group (Figure 4A). All tissue types surpassed the threshold for most animals in the 10 mg/kg treatment group at 1- and 4 h post treatment (Figure 4B). Liver and spleen remained in excess of the threshold for several animals at the 24 h time point.

#### Fat

**Figure 5:**
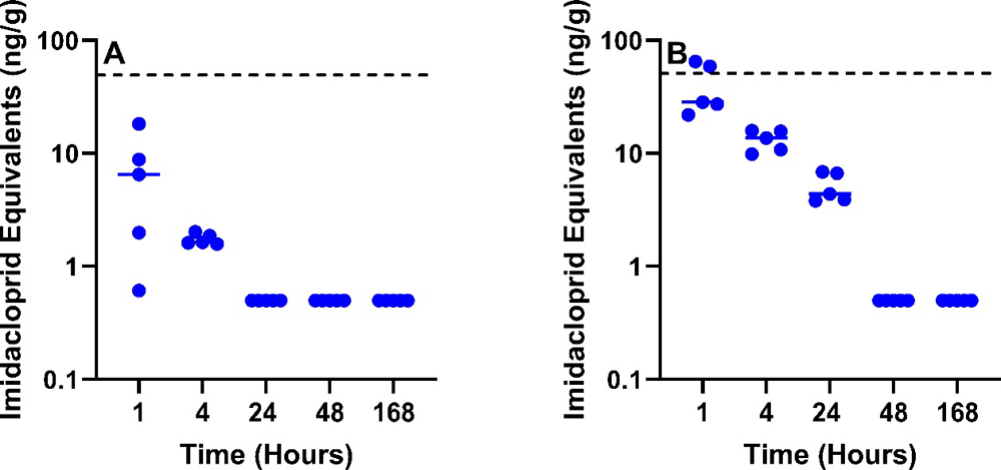
Sum of IMI and molar equivalents of 6-chloropyridinyl moiety metabolites for poultry fat. IMI dosing of poultry at (A) 1 mg/kg and (B) 10 mg/kg. The dashed line represents the EFSA 396/2005 and 40 CFR § 180.472 tolerance thresholds for poultry fat (50 ng/g).

While IMI was present in adipose tissue at 1 and 4 h post-treatment in the 1 mg/kg group, no animal exceeded the 50 ppb (50 ng/g) threshold (Figure 5A). Within the 10 mg/kg treatment group, 2 of 5 animals exceeded the threshold at 1 h post treatment (Figure 5B).

#### Eggs

**Figure 6:**
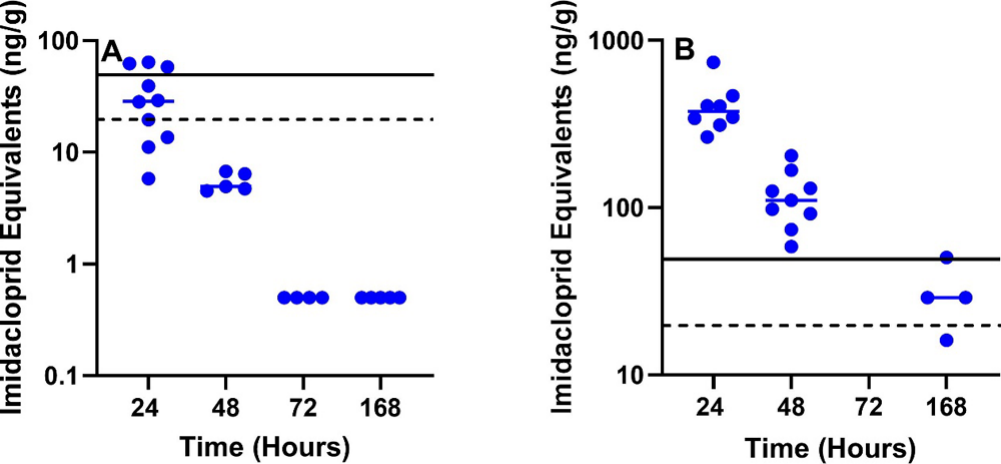
Sum of IMI and molar equivalents of 6-chloropyridinyl moiety metabolites for poultry eggs. IMI dosing of poultry at (A) 1 mg/kg and (B) 10 mg/kg. Dashed line represents the 40 CFR § 180.472 tolerance threshold for poultry eggs(20 ng/g) and the solid line for EFSA 396/2005 (50 ng/g).

In the 1 mg/kg treatment group, 6 of 10 eggs collected 24 h post-treatment exceeded the CFR regulatory threshold of 20 ppb (20 ng/g) (Figure 6A). There were no detectable concentrations at 72 h (3d) and beyond. On the contrary, the 10 mg/kg treatment group still had 3 of 4 eggs exceeding 20 ppb at 168 h (7 days) postexposure (Figure 6B).

## Discussion

By 2025, more than 90% of the commercial egg producing facilities in the United States are predicted to be cage-free.^20^ Given welfare considerations to enrich the layers’ environment, the use of litter is considered to be an appropriate provision to enable hens to satisfy their ethological needs.^21^ Although the makeup of the litter has not been defined, wood shavings and sawdust have been historically used by the U.S poultry industry.^22^ While IMI is not currently used in the layer industry, the transition to cage-free environments may prompt its consideration for pest control.

Like Bean et al., we first sought to examine the temporal profile of low and high doses of IMI in poultry that could be achieved by chemical application in house litter.^7^ The LD50 in layers has been reported at 104.1 mg/kg.^14^ As IMI stimulates nicotinic acetylcholine receptors in the central nervous system, high doses of IMI have the potential to cause neurologic signs. In the Roy et al. study, birds given 1 mg/kg showed no outward clinical signs, whereas moderate sedation occurred within minutes of 10 mg/kg IMI administration but resolved.^8^ In another study, the adjusted effective dose (ED50) for neurologic signs in chickens was 11.2 mg/kg/day.^23^ In this study, birds dosed with either 1 or 10 mg/kg IMI did not show any neurologic signs (depression, muscle tremors, ataxia, or seizures) at any point during the study. Furthermore, no gross lesions were observed at post-mortem examination in any of the birds, which emphasizes the fact that subclinical dosing of IMI can be clinically and grossly inapparent.

The absorption, distribution, and metabolism profile observed in this study was similar to that described by Bean et al., with rapid absorption, distribution, metabolism, and excretion occurring within 24 h.^7^ However, there were several unique differences. In the current study, IMI-olefin was the most abundant metabolite observed in all sampled tissues except the kidney. Also, IMI-urea was present in most tissues and was as abundant as 5-OH-IMI in the kidney. This finding was not too surprising given that the hydrolysis of IMI to IMI-urea is intended to facilitate its excretion from the body in urine/uric acid. Additionally, we observed the presence of 6-OH-NA in fecal matter collected at 24 and 48 h post treatment. This is a novel find as an excretory product from an animal. It is unclear whether it was derived strictly from the bird’s intestinal microflora or not. This may require further exploration but is not germane to the assessment of food safety, as this metabolite was not observed in edible tissues. Of additional interest was the finding of IMI-olefin, 5-OH-IMI, and IMI-urea metabolites in eggs within 24 h postexposure at concentrations exceeding current regulatory thresholds. This has clear and serious implications for food safety.

Food safety is a concern when pesticides are introduced into an environment in which production animals reside. As such, the EFSA 396/2005 and Code of Federal Regulations (40 CFR § 180.472) define tolerances for a variety of agricultural products to IMI and its metabolites.^12^ For IMI, compliance is determined by measuring the sum of imidacloprid and its metabolites containing the 6-chloropyridinyl moiety, calculated as the stoichiometric equivalent of IMI.^12^ For poultry, tolerances of 50 ng/g (ppb) have been assigned to meat (muscle), meat byproducts (brain, liver, spleen, kidney, and other edible tissue), and fat. For eggs, a tolerance of 20 ng/g (ppb) was assigned. From this study, there is clear evidence that the sum of IMI equivalent concentrations exceeds the regulatory threshold for meat, poultry byproducts, fat, and eggs following treatment with a high dose of IMI. There is additional concern for eggs derived from birds with low dose exposures.

Imidacloprid is moderately water-soluble (610 mg/L at 20 °C), so it is not expected to accumulate in the egg yolk, which contains 99% of an egg’s fat in comparison to more lipophilic pesticides like organochlorine pesticides.^5^ This study did not distinguish the IMI distribution between egg whites and yolks, as the entire edible egg is considered the subject for adherence to regulatory thresholds. However, we identified a significant accumulation of IMI and its metabolites being introduced into fully formed eggs up to 7 days postexposure from clinically healthy hens.

In conclusion, application of IMI to layer houses poses an undue risk to food safety. Given the absence of outward signs of neurotoxicity to the animals used in this study, the potential for pesticide accumulation in edible tissues and other poultry products with prolonged exposure to low-concentration treatments is possible. Given these concerns, a withdrawal time must be established for edible tissues for which tolerance values have been established in the United States or MRLs in the European Union. Unlike medicated drug use and exposure, the Food Animal Residue Avoidance Database (FARAD) does not address pesticide exposure in production animals. Further work is underway to address chronic, low dose IMI exposure risks to layers and their egg products. The culmination of this work may help guide the development of food safety-specific application rates for IMI in poultry houses and assist with establishing withdrawal times from houses in which the pesticide has been misapplied.

## Supporting Information Available

Additional results, IMI and its metabolites over time in meat, in adipose tissue, in meat byproducts, in eggs, and in feces, and the range of percent recoveries of IMI and its metabolites in the different tissue types (PDF)

## ABBREVIATIONS

5-OH-IMI: 5-hydroxyimidacloprid
6-CNA: 6-chloronicotinic acid
6-OH-NA: 6-hydroxy nicotinic acid
CFR: Code of Federal Regulations
EFSA: European Food Safety Authority
IMI: Imidacloprid
LC-MS/MS: Liquid Chromatography Tandem Mass Spectrometry
LD50: Median Lethal Dose
MRM: Multiple Reaction Monitoring
PPB: Parts Per Billion
