# Supplementary material for: Temporal Distribution of Imidacloprid and Its Metabolites in Laying Hens: An Evaluation of the Potential Impact on Food Safety
Source: J Agric Food Chem. 2025 May 2;73(19):11941–7. doi: 10.1021/acs.jafc.4c08378 (PMC12082688; doi:10.1021/acs.jafc.4c08378)
Supplement: Supplementary file 1 — jf4c08378_si_001.pdf [file jf4c08378_si_001.pdf]

Temporal Distribution of Imidacloprid and Its Metabolites in Laying Hens: An Evaluation of the Potential Impact on Food Safety

Mayra F. Tsoi[1*], Justin Zyskowski [2], Cara Robison [3], Andreas F. Lehner [2], Levent Dirikolu [4], and John P. Buchweitz [1]

1. Department of Pathobiology and Diagnostic Investigation, College of Veterinary Medicine, Michigan State University, 784 Wilson Rd, East Lansing MI 48824
2. Veterinary Diagnostic Laboratory, Michigan State University, 4125 Beaumont Rd, Lansing MI 48910
3. Department of Animal Sciences, Michigan State University, 474 Shaw Lane, East Lansing MI 48824
4. Department of Comparative Biomedical Sciences, School of Veterinary Medicine, Louisiana State University, Skip Bertman Dr, Baton Rouge LA 70803

***Corresponding author:** Mayra F. Tsoi, Veterinary Diagnostic Laboratory, Michigan State University, 4125 Beaumont Road, Lansing, MI 48910-8104, USA, email: tsoimayr@msu.edu (517) 432-6290

Supporting Information

SUPPLEMENTAL FIGURES

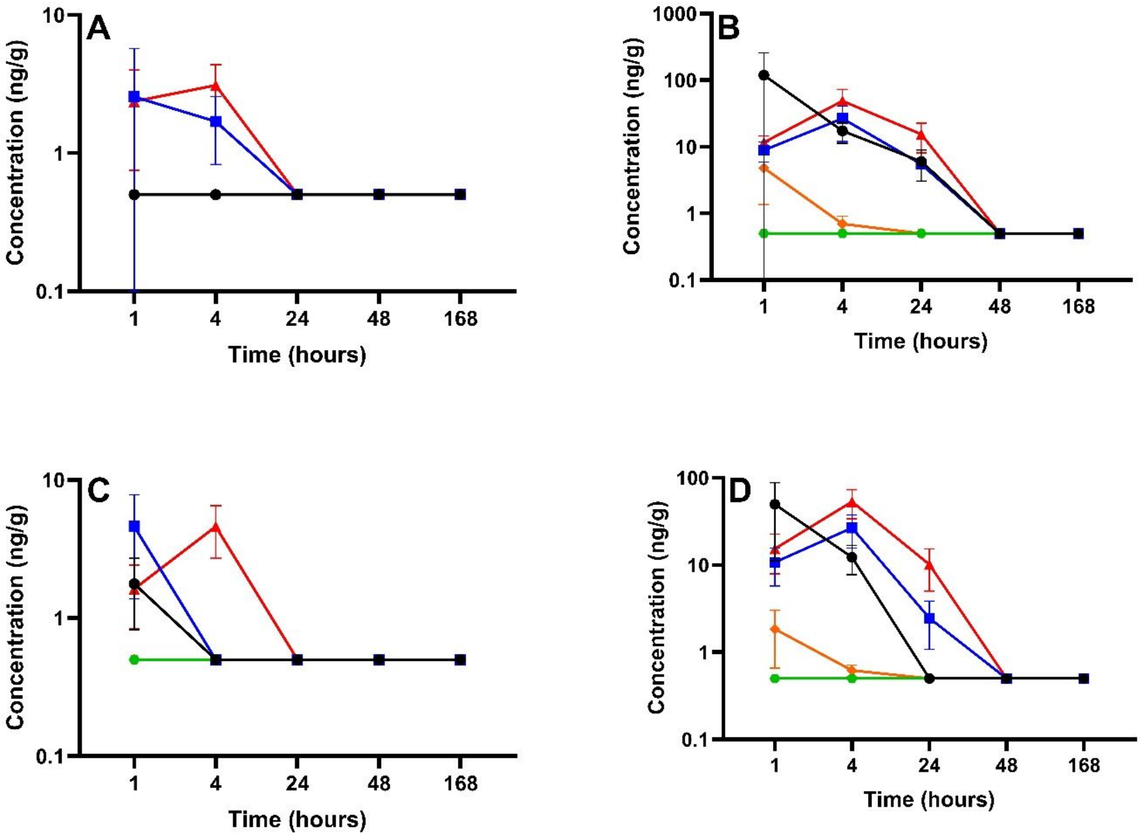

Supp. Figure 1. Imidacloprid and its metabolites in pectoral and thigh muscle (meat). IMI dosing of poultry at (A, C) 1 mg/kg and (B, D) 10 mg/kg. IMI (black), 5-OH-IMI, (blue), IMI-olefin (red), IMI-urea (orange), desnitro-IMI (green), and 6-CNA (purple) were measured in pectoral (A, B) and thigh muscle (C, D) by LC-MS/MS to an LOQ of 0.5 ng/g (ppb). Results are presented as mean ± standard deviation.

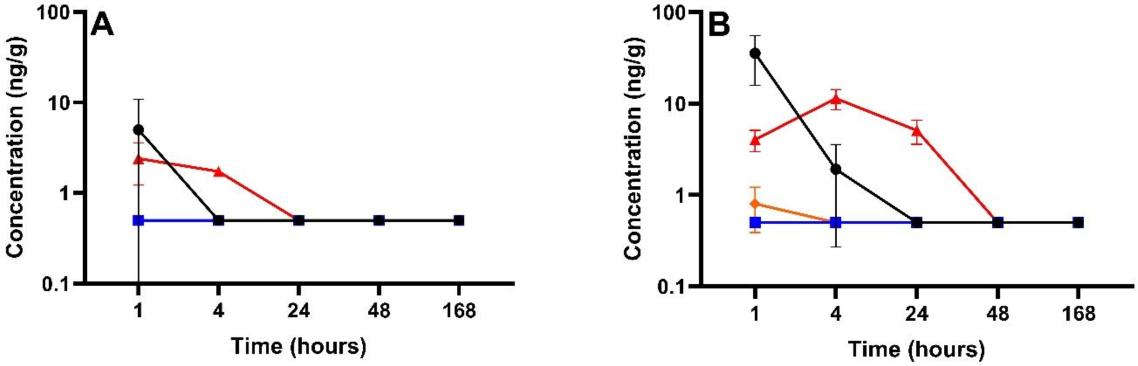

Supp. Figure 2. Imidacloprid and its metabolites in adipose tissue. IMI dosing of poultry at (A) 1 mg/kg and (B) 10 mg/kg. IMI (black), 5-OH-IMI, (blue), IMI-olefin (red), IMI-urea (orange), desnitro-IMI (green), and 6-CNA (purple) were measured in fat by LC-MS/MS to an LOQ of 0.5 ng/g (ppb). Results are presented as mean ± standard deviation.

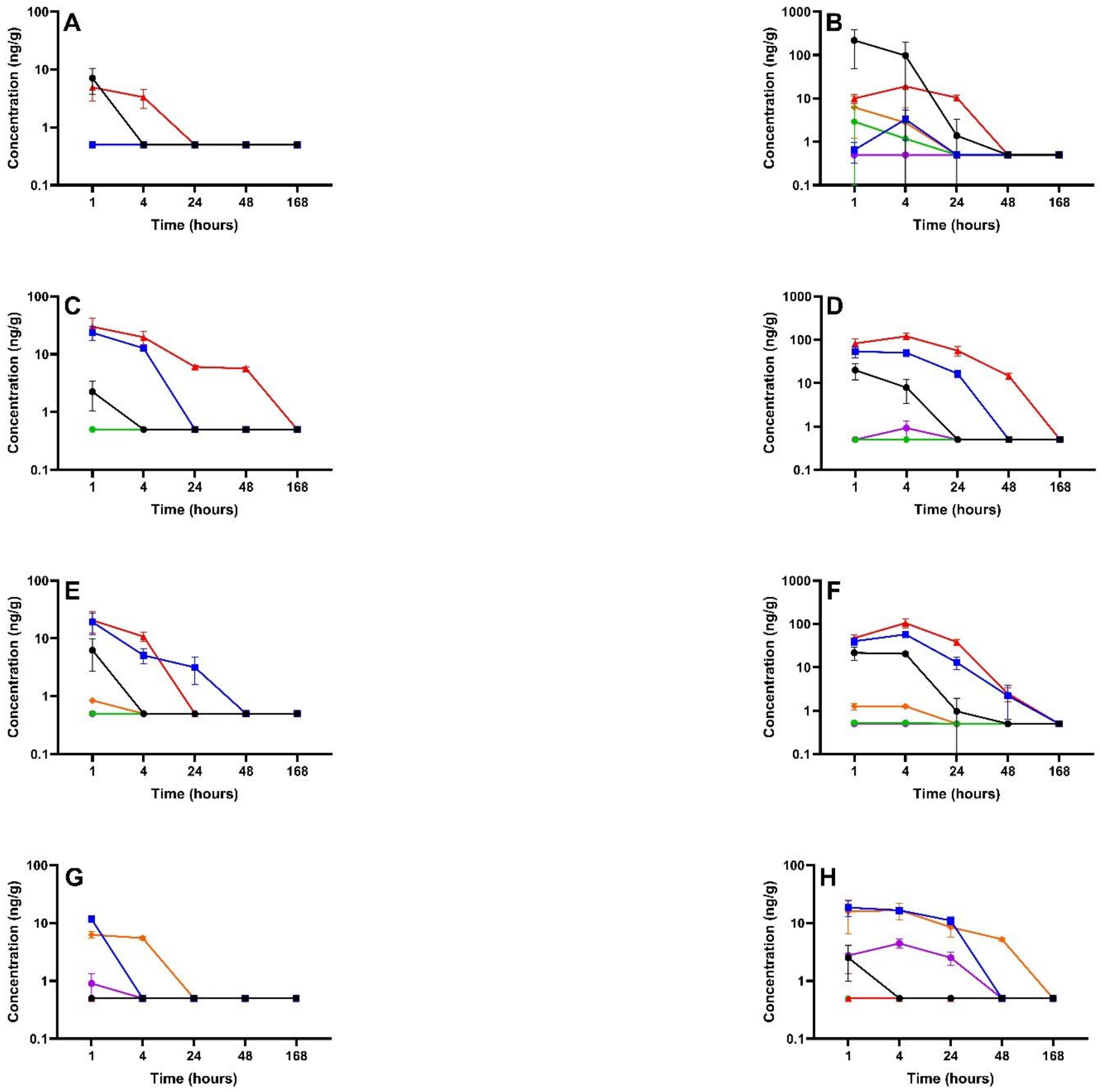

Supp. Figure 3. Imidacloprid and its metabolites in meat byproducts [brain (A, B), liver (C, D), spleen (E, F), and kidney (G, H)]. IMI dosing of poultry at (A, C, E, G) 1 mg/kg and (B, D, F, H) 10 mg/kg. IMI (black), 5-OH-IMI, (blue), IMI-olefin (red), IMI-urea (orange), desnitro-IMI (green), and 6-CNA (purple) were measured in plasma by LC-MS/MS to an LOQ of 0.5 ng/mL (ppb). Results are presented as mean ± standard deviation.

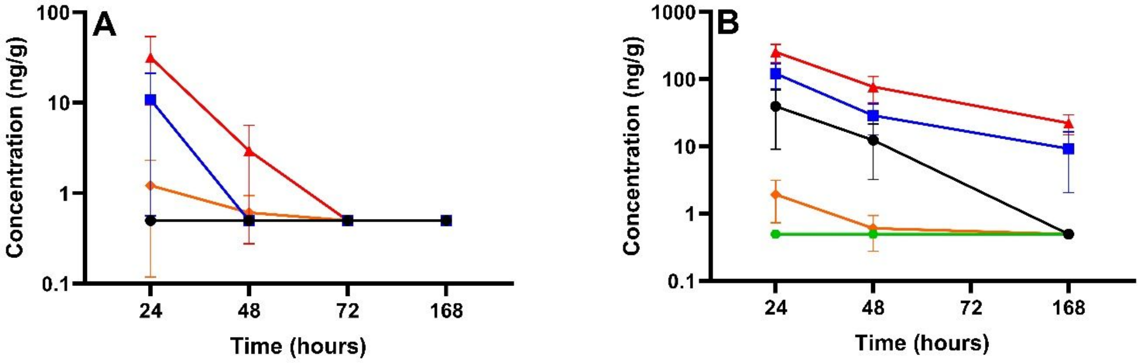

Supp. Figure 4. Imidacloprid and its metabolites in egg at (A) 1 mg/kg and (B) 10 mg/kg. IMI (black), 5-OH-IMI, (blue), IMI-olefin (red), IMI-urea (orange), desnitro-IMI (green), and 6-CNA (purple) were measured in eggs by LC-MS/MS to an LOQ of 0.5 ng/g (ppb). Results are presented as mean ± standard deviation.

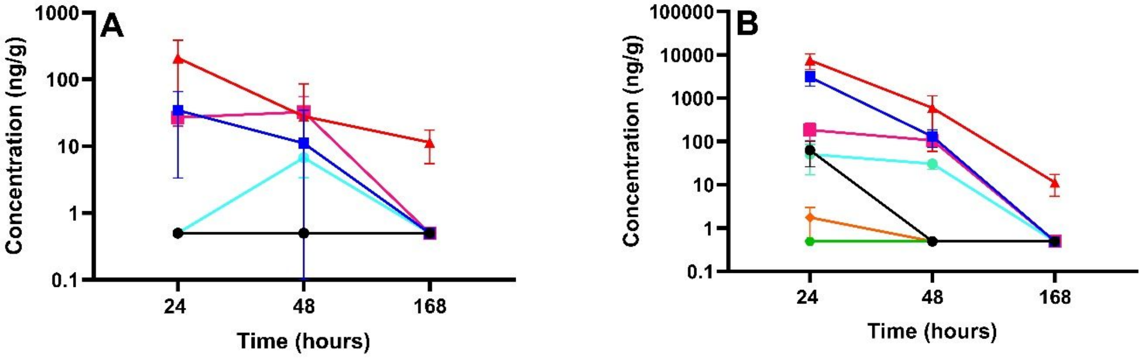

Supp. Figure 5. Imidacloprid and its metabolites in feces at (A) 1 mg/kg and (B) 10 mg/kg. IMI (black), 5-OH-IMI, (blue), IMI-olefin (red), IMI-urea (orange), desnitro-IMI (green), 6-CNA (purple), and 6-OH-NA (pink) were measured in feces by LC-MS/MS to an LOQ of 0.5 ng/g (ppb). Results are presented as mean ± standard deviation.

SUPPLEMENTAL TABLE

Supp Table 1: Range of percent recoveries of IMI and its metabolites in tissue matrices.

	Plasma1	Brain1	Liver1	Kidney1	Spleen1	Muscle	Egg1
IMI	96-116%	89-117%	93-110%	91-113%	92-111%	92-114%	89-112%
5-OH-IMI	90-147%	92-141%	97-146%	80-139%	89-154%	88-146%	91-138%
IMI-urea	101-112%	93-107%	99-115%	90-112%	97-114%	99-120%	92-112%
IMI-olefin	96-118%	93-125%	96-115%	89-109%	95-114%	96-111%	93-117%
Desnitro-IMI	91-125%	94-115%	94-115%	88-109%	90-116%	96-121%	98-119%
6-CNA	94-116%	93-118%	102-115%	88-116%	94-118%	100-119%	99-117%
6-OH-IMI	94-134%	94-128%	99-137%	63-159%	94-133%	94-132%	102-132%
